# Factors Influencing the Association Between Coach and Athlete Rating of Exertion: a Systematic Review and Meta-analysis

**DOI:** 10.1186/s40798-020-00287-2

**Published:** 2021-01-05

**Authors:** Darren Paul, Paul Read, Abdulaziz Farooq, Luke Jones

**Affiliations:** 1 Research and Scientific Support, Aspetar – Qatar Orthopaedic and Sports Medicine Hospital, PO BOX 29222, Doha, Qatar; 2 Faculty of Health Sciences, University of Hull, Hull, HU6 7RX UK

**Keywords:** Rate, Perceived, Intended, Exertion, Sport

## Abstract

**Background:**

Subjective monitoring of rate of perceived exertion is common practice in many sports. Typically, the information is used to understand the training load and at times modify forthcoming sessions. Identifying the relationship between the athlete and coach’s interpretation of training would likely further benefit understanding load management. The aim of this systematic review was to evaluate the relationship between coaches’ rating of intended exertion (RIE) and/or rating of observed exertion (ROE) and athletes’ reported rating of perceived exertion (RPE).

**Methods:**

The review was undertaken in accordance with the Preferred Reporting Items for Systematic Reviews and Meta-Analyses guidelines. We conducted a search of Medline, Google Scholar, Science Direct, SPORTDiscus, and Web of Science databases. We assessed the correlation between coach-reported RIE and/or ROE and RPE. Assessment for risk of bias was undertaken using the Quality Appraisal for Reliability Studies (QAREL) checklist. Inclusion criteria were (1) male and/or female individuals, (2) individual and/or team sport active participants, and (3) original research article published in the English language.

**Results:**

Data from 19 articles were found to meet the eligibility criteria. A random effect meta-analysis based on 11 studies demonstrated a positive association of player vs. coach rating of RIE (*r* = 0.62 [95% CI 0.5 to 0.7], *p* < 0.001). The pooled correlation from 7 studies of player vs. coach rating on ROE was *r* = 0.64 95% CI (0.5 to 0.7), *p* < 0.001.

**Conclusion:**

There was a moderate to high association between coach RIE and/or ROE and athlete-reported RPE and this association seems to be influenced by many factors. The suggestions we present in this review are based on imploring practitioners to consider a multi-modal approach and the implications of monitoring when using RPE.

**Trial Registration:**

CRD42020193387

## Key Points

- The agreement between coach and athlete reporting of exertion is generally moderate to good but can vary among studies.
- Practitioners should be aware of multiple possible factors that may impact the association between coaches’ and athletes’ rating of exertion.
- Practitioners should develop strategies to improve understanding and develop relations to enhance the effective implementation of RPE monitoring.

## Background

### Load Monitoring

The practice of load monitoring is now common within many team and individual sports. The primary objectives are to improve athlete readiness for training and minimize the risk of non-functional overreaching, injury, and/or illness [1]. A mixture of subjective and objective methods are used to quantify the internal and external demands [2, 3]. Subjective measures have been shown to reflect acute and chronic training-related changes in athletes and may trump objective measures for monitoring training response [4]. Though subjective measures of load monitoring are often used in the applied setting, it should be recognized that there may be threats to the quality of the data received by the practitioner.

Session rating of perceived exertion (RPE) is commonly used to measure internal load and is based on the calculation of athletes’ rate of score on the Borg category 1–10 scale multiplied by the exercise duration [5]. The popularity of RPE is fortified by evidence showing it to be valid, reliable, and sensitive in a range of contexts, with the benefit of being easy to administer and inexpensive [6]. The athlete’s response can provide instantaneous feedback to the sports science, medical, and coaching staff to inform decision-making and help determine the athletes’ state of readiness to train/play. Also, coaches can use this feedback to ascertain whether the athletes’ reported RPE is aligned with their own rating of intended (RIE) (pre-training/competition), or rating of observed exertion (ROE) (post-training/competition).

Session rate of perceived exertion is considered a biopsychosocial construct; however, it is not always represented this way in an applied setting. Anecdotally, fitness practitioners may gravitate towards physical elements of load monitoring given the practitioners’ role and area of knowledge. Identifying a potential mismatch between a coach and athletes’ rating of exertion for any given training session or match is worthwhile since training load errors may manifest in poor load management and/or reduced performance. Accordingly, the objectives of this review were to (1) examine the relationship between athletes’ RPE and coaches’ RIE and/or ROE; (2) identify possible contributing factors that may explain a potential relation, notably the effects of different sports, seasonal phases, exercise selection, classification, age, fitness, coaching experience, co-observer, and scale used; and (3) propose strategies that may enhance the relationship between coach and athletes’ reported rating of exertion.

## Methods

### Eligibility Criteria

Eligibility criteria for study inclusion consisted of one of the following: (1) study reported correlation of coaches’ RIE and/or ROE with athletes’ RPE; (2) athletes performing team or individual sports. In addition, the reference lists of retrieved full-text articles and recent reviews were examined to identify articles not found by our initial search.

### Search

A systematic review of all published literature was undertaken in accordance with the Preferred Reporting Items for Systematic Reviews and Meta-Analyses (PRISMA) guidelines [7]. Two researchers independently searched the electronic databases PubMed, Google Scholar, SPORTDiscus, Science Direct, and Web of Science using the following keywords: rating, rate, perceived, perception, intended, observed, actual, exertion, effort, athlete, coach, sport, team, individual, wellness, subjective, scale, monitoring, internal, external, load, management, training, match, overload, competition.

### Search Methods and Study Selection

**Fig. 1:**
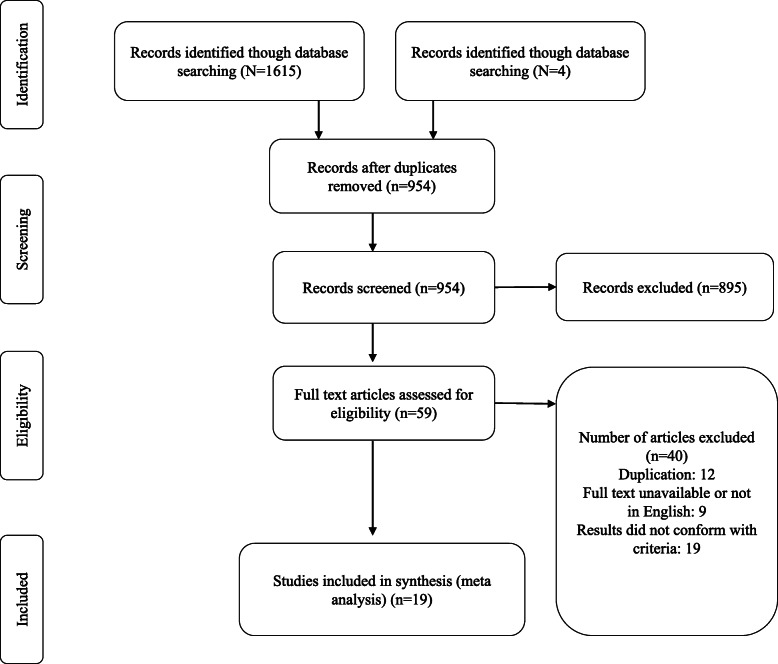
Schematic representation of process and reason for selection for the studies included

Selection of studies was conducted in two consecutive phases (Fig. 1). Phase one consisted of screening for (1) duplicates, (2) title, and (3) abstract. The second phase involved screening the full manuscript using the inclusion criteria. Studies were included if they fulfilled the following criteria: (1) written in English, (2) full-text articles published in peer-reviewed journals, (3) included coach RIE/ROE as well as athlete RPE and correlation coefficient was reported. The time period of literature selection included studies published up until June 2019.

### Data Collection

Two researchers independently screened and extracted data from each source document which included study identification information, the number of participants, demographic information (including the sex, age, and standard of play), sporting discipline, monitoring tool used, coaches’ RIE/ROE, athletes’ RPE, coaches’ observed scoring, correlation relationship, and factors that may contribute to the reported scores.

### Risk of bias

Two authors independently assessed the risk of bias for each study according to the criteria of the Cochrane Handbook for Systematic Reviews of Interventions (Table 1). Each study was analyzed for random sequence generation, allocation concealment, blinding of participants and personnel, blinding of outcome assessment, incomplete outcome data, selective reporting, and other biases; the risk of bias arising from each domain is judged as “low,” “high,” or “unclear.” Further analysis was undertaken using the Quality Appraisal for Reliability Studies (QAREL) checklist [8] (Table 2). Each item on QAREL can be answered “yes,” “no,” or “unclear.” In addition, some items include the option “not applicable.” The questions have been worded so that a “yes” response indicates a good-quality aspect of the study, whereas a “no” response indicates a poor-quality aspect.

**Table 1:**
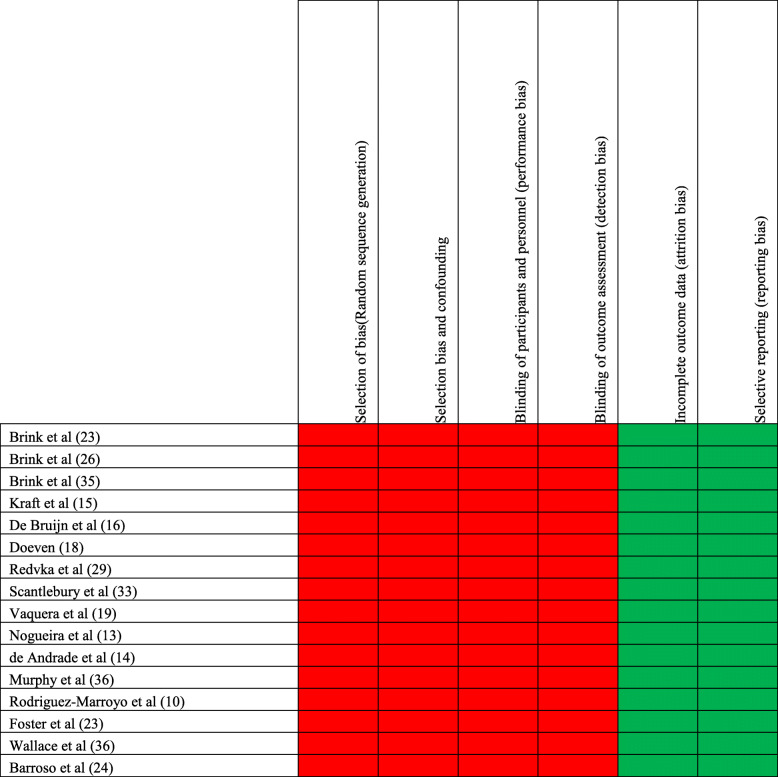
Risk of bias for selection of studies

Green = low risk of bias

Red = high risk of bias

**Table 2:** Results of the QAREL checklist

Item		Brink et al [9]	Kraft et al [10]	Barnes et al [11]	De Bruijn et al [12]	Doeven [13]	Redvka et al [14]	Scantlebury et al [15]	Vaquera et al [16]	Brink et al [17]	Rabelo et al [18]	Andrade Nogueira et al [19]	de Andrade et al [20]	Barroso et al [21]	Murphy et al [22]	Rodriguez-Marroyo et al [23]	Brink et al. [24]	Viveiros et al [25]	Wallace et al [26]	Foster et al [27]
1	Was the test evaluated in a sample of subjects who were representative of those to whom the authors intended the results to be applied?	Y	Y	Y	Y	Y	Y	Y	Y	Y	Y	Y	Y	Y	Y	Y	Y	Y	Y	Y
2	Was the test performed by raters who were representative of those to whom the authors intended the results to be applied?	Y	Y	Y	Y	Y	Y	Y	Y	Y	Y	Y	Y	Y	Y	Y	Y	Y	Y	Y
3	Were raters blinded to the finding of other raters during the study?	Y	Y	Y	Y	Y	Y	Y	Y	Y	Y	Y	Y	Y	Y	Y	Y	Y	Y	Y
4	Were raters blinded to their own prior findings of the test under evaluation?	N	N	N	N	N	N	N	N	N	N	N	N	N	N	N	N	N	N	N
5	Were raters blinded to the results of the accepted reference standard or diseased status for the target disorder (or variable) being evaluated?	NA	NA	NA	NA	NA	NA	NA	NA	NA	NA	NA	NA	NA	NA	NA	NA	NA	NA	NA
6	Were raters blinded to clinical information that was not intended to be provided as part of the testing procedure or study design?	N	N	N	N	N	N	N	N	N	N	N	N	N	N	N	N	N	N	N
7	Were raters blinded to additional cues that were not part of the test?	N	N	N	N	N	N	N	N	N	N	N	N	N	N	N	N	N	N	N
8	Was the order of examination varied?	Unclear	Unclear	Unclear	Unclear	Unclear	Unclear	Unclear	Unclear	Unclear	Unclear	Unclear	Unclear	Unclear	Unclear	Unclear	Unclear	Unclear	Unclear	Unclear
9	Was the stability (or theoretical stability) of the variable being measured taken into account when determining the suitability of the time interval between repeated measures?	Y	Y	Y	Y	Y	Y	Y	Y	Y	Y	Y	Y	Y	Y	Y	Y	Y	Y	Y
10	Was the test applied correctly and interpreted appropriately?	Y	Y	Y	Y	Y	Y	Y	Y	Y	Y	Y	Y	Y	Y	Y	Y	Y	Y	Y
11	Were appropriate statistical measures of agreement used?	Y	Y	Y	Y	Y	Y	Y	Y	Y	Y	Y	Y	Y	Y	Y	Y	Y	Y	Y

### Synthesis of Results

The estimate of the pooled correlation coefficients extracted from the included studies was combined in a meta-analysis, with a random effect model chosen since included studies used different tools, involving athletes from various sports, and sport type (team vs. individual) (Table 3). Heterogeneity was determined using the *I*^2^ statistics. An *I*^2^ of 0–40% was classified as low heterogeneity and 75–100% considerable heterogeneity [28]. Publication bias was estimated using funnel plots (a scatter plot of Fisher’ *Z* and standard errors from each study) and asymmetry was diagnosed based on visual inspection. Comprehensive Meta-Analysis version 3.3 (Biostat, Englewood, NJ, USA) was used for performing the meta-analysis [29].

**Table 3:** Summary of included studies reporting coach-intended and/or observed and athlete-reported rating of perceived exertion

***Reference***	Participants characteristics				Analysis			Results						
				Coach details	Training content	Scale	Metrics	Coaches		Coaches	Athletes			
	*Age*	*Status/level*	*Sport*	*Number of coaches and/or yrs experience*				***RIE***		***ROE***	***RPE***	***RIE vs. RPE***	***ROE vs. RPE***	Other
Brink et al [9]	12 M (20.3 ± 1.5 yrs)	Professional Club	Soccer	1 coach 13 yrs experience	26 training sessions (2 4-week mesocycles)	6-20 Borg scale	No Feedback	15.0 ± 1.4		15.1 ± 1.5	15.0 ± 1.9			Difference between ROE-RPE feedback (F(1.229) = 9.16, *p* = 0.003, ES 0.4)
					231 exertion scores		Feedback	14.8 ± 1.8		15.2 ± 2.0	14.8 ± 2.4			
Kraft et al [10]	56 M & F	Intercollegiate	F soccer, volleyball and basketball	4 coaches	433 training sessions	CR10						*sRIE CI vs. RPE*	*sROE vs. RPE*	sRIE vs. sROE
			M basketball				sRPE Overall	620 ± 322		575 ± 312	513 ± 300	*r* = 0.65	*r* = 0.83	*r* = 0.93
					Volleyball = 74		sRPE Volleyball	555 ± 177		589 ± 186	494 ± 198	*r* = 0.31	*r* = 0.32	*r* = 0.91
					Socce*r* = 123		sRPE Soccer	240 ± 175		04 ± 164	219 ± 175	*r* = 0.79	*r* = 0.72	*r* = 0.89
					Womens’ basketball = 124		sRPE Womens Basketball	815 ± 245		763 ± 255	711 ± 282	*r* = 0.71	*r* = 0.60	*r* = 0.83
					Mens’ basketball = 112		sRPE Mens basketball	864 ± 130		760 ± 165	628 ± 220	*r* = 0.42	*r* = 0.48	*r* = 0.74
					Hard intensity			6.5 ± 1.2		8.2 ± 0.4			*r* = -0.26	
					Moderate intensity			6.1 ± 1.2		5.6 ± 0.7			*r* = 0.28	
					Easy intensity			3.7 ± 1.9		2.9 ± 1.1			*r* = 0.58	
Barnes et al [11]	13 M (20.2 ± 1.4 yrs)	Highly trained	Cross-country runners	2 coaches	110 training days (3024 sessions)	CR10	Season average							
	12 F (19.7 ± 1.6 yrs)						sRPE load (F)							
							Easy intensity	64 ± 12			92 ± 53			
							Moderate intensity	244 ± 34			259 ± 87			
							Hard intensity	551 ± 84			507 ± 118			
							sRPE load (M)							
							Easy intensity	82 ± 21			157 ± 88			
							Moderate intensity	351 ± 46			448 ± 149			
							Hard intensity	646 ± 105			658 ± 157			
De Brujin et al [12]	9 (7 M & 2 F) 22 ± 3 yrs	Amateur	Soccer	2 physiotherapists with “multiple years of experience” and involved in athletes’ rehabilitation for 6 months and with “several years” outdoor rehabilitation training experience	3 training sessions	6 to 20 Borg scale						*RIE vs. RPE*		*RIE vs. Distance*
							Overall					*r* = 0.35		*r* = 0.26
							Warmup					*r* = 0		*r* = 0.30
							Core 1					*r* = 0.61		*r* = 0.65
							Core 2					*r* = 0.75		*r* = -0.08
Doeven et al [13]	14 M (26.7 ± 3.8 yrs)	Elite	Basketball	1 coach (<10 yrs)	Competition (8 domestic, 1 cup and 6 euro leagues ) within 6 weeks (2.5 matches per week)	6 to 20 Borg scale								
							RPE	16.1 ± 1.4			15.6 ± 2.3			
							sRPE load	418 ± 130			403 ± 135	*r* = 0.25; *p* < 0.01		
Redvka et al [14]	24 M (24.1 ± 3.4 yrs)	Professional	Soccer	2 coaches	22 training sessions (first 3 weeks of pre-season)	CR10		6.7 ± 1.78			6.8 ± 1.43	*r* = 0.60; *p* = 0.003; ES = 0.05		
				1 technical coach (8 yrs coaching experience)	1465 mins									
				1 Physical coach (20 yrs experience)										
Scantlebury et al [15]	9 F (17.4 ± 0.8 yrs)	Independent school	Hockey	4 coaches (one per sport). All coaches had > 5 yrs and had worked with participants for > 1 yr	Training 28, 125, and 66 easy, moderate, and hard training sessions were analyzed	CR10						*RIE vs. RPE*		*RIE vs. ROE*
	8 F (17.6 ± 0.6 yrs)		Netball				Overall	3.6 ± 1.2		3.5 ± 1.1	3.5 ± 1.8	*r* = 0.39		*r* = 0.63
	10 M (17.2 ± 0.4 yrs)		Rugby union				Easy intensity	1.9 ± 0.3		2.3 ± 0.9	3.8 ± 2.2	*r* = 0.39; 95%CI 0.02-0.67		*r* = 0.54 95% CI 0.09-0.76
	10 M (17.2 ± 0.8 yrs)		Soccer				Moderate intensity	3.2 ± 0.4		3.1 ± 0.4	2.9 ± 1.2	*r* = 0.27; 95% CI 0.1-0.43		*r* = 0.20 95%CI 0.02-0.36
							Hard intensity	5.2 ± 0.6		4.6 ± 1.1	4.5 ± 2.1	*r* = 0.46; 95% CI 0.25-0.63		*r* = 0.79; 95%CI 0.68-0.87
Vaquera et al [16]	12 M (16 ± 0.4 yrs)	Junior National League	Basketball	1 coach with 18 yrs experience training U16 and U18	Training (6 weeks)	CR10	9 training sessions							
							7 games 1vs1 SSG		No data					
							6 games 2 vs. 2 SSG							
							8 games of 5 vs. 5 SSG							
							5 games of 3 vs. 2 SSG							
Brink et al [17]	31 M	Highest level	Soccer	1 U15 coach = 18 yrs experience	977 training sessions (8 weeks)	6 to 20 Borg scale		13.3 ± 2.1		13.3 ± 2.2	13.6 ± 2.2	*RIE vs*. *RPE*	*ROE vs. RPE*	
	16 (14.3 ± 0.3 yrs)	competition		1 U17 coach = 23 yrs experience	u15 = 445 sessions							(*r* = 0.58 (*p* < 0.01))	(*r* = 0.64 (*p* < 0.01))	
	17 (16.3 ± 0.2 yrs)				u17 = 532 session									
Rabelo et al [18]	18 M (24.6 ± 3.8 yrs)	Professional	Futsal	1 coach with 3 yrs experience with tool	314 training sessions (45 weeks)	CR10								
							Low							
							Pre-season	3.9 ± 1.2			2.9 ± 0.5			
							Comp 1	3.3 ± 0.5			3.0 ± 0.6			
							Inter Comp	nr			nr			
							Comp 2	3.0 ± 1.0			3.7 ± 0.8			
							Moderate							
							Pre-season	5.3 ± 0.7			4.7 ± 0.6			
							Comp 1	4.9 ± 0.5			4.3 ± 0.5			
							Inter Comp	5.5 ± 0.5			4.8 ± 0.6			
							Comp 2	6.4 ± 0.5			4.2 ± 0.3			
							High							
							Pre-season	7.6 ± 0.5			6.0 ± 0.9			
							Comp 1	6.4 ± 0.6			5.1 ± 0.4			
							Inter Comp	7.6 ± 0.5			5.4 ± 0.6			
							Comp 2	7.5 ± 0.8			6.0 ± 0.4			
Andrade Nogueira et al [19]	17 (10 M, 7F) (15.2 ± 0.57 yrs)	State and National	Swimmers	1 coach	18 training sessions	CR10	All sessions	3.4 ± 1.2			3.4 ± 1.9	ICC = 0.80; 95%CI = 0.75 to 0.84; *p* = 0.001		
							Transformation phase	3.9 ± 1.4			4.2 ± 2.0	ICC = 0.79; 95%CI = 0.71 to 0.84; *p* = 0.001		
							Taper phase	2.9 ± 0.3			2.2 ± 0.8	ICC = 0.19; 95%CI = − .10 to 0.41; *p* = 0.001		
de Andrade et al [20]	15 M (24 ± 2.8 yrs)	National Championship	Volleyball	1 coach	34 training sessions (510 data points)	CR10	Total					k = 0.64		
							Setter					*k* = 0.78		
							Liberos					*k* = 0.79		
							Outside hitters					*k* = 0.75		
							Middle blockers					*k* = 0.74		
							Opposites					*k* = 0.75		
Barroso et al [21]	160	Local, State and National level	Swimmers	9 coaches		CR10	All age groups					*r* = 0.60; *p* < 0.001		
	11-12 yrs *n* = 46						11–12 yrs					*r* = 0.31; *p* < 0.001		
	13–14 yrs *n* = 65						13–14 yrs					*r* = 0.51; *p* < 0.001		
	15–16 yrs *n* = 49						15–16 yrs					*r* = 0.74; *p* < 0.001		
Murphy et al [22]	14 (8 M & 6 F) (15 ± 1.2 yrs)	Elite	Tennis	6 coaches (10 ± 3 yrs coaching)	21 ± 3 training sessions (16 weeks, 285 drills )	CR10								*RIE vs. ROE*
						Mental exertion (0–10)	predicted sRPE	5.5 ± 1.2						ICC = 0.79
							session RPE	5.4 ± 1.1			6.2 ± 1.4	*r* = 0.59		
							Drill Physical sRPE	5.1 ± 1.7			5.4 ± 1.7	*r* = 0.71		
							Drill Mental sRPE	6.0 ± 1.6			5.8 ± 1.6	*r* = 0.68		
Rodrguez-Marrayo et al [23]	12 F (21 ± 3 yrs)	University	Volleyball	4 coaches	15 weeks of training	CR10		*Expert coach*	*Beginner coach*					
				2 experts (< 10 yrs)			sRPE—mean weekly	231 ± 66	228 ± 64		245 ± 72			
				2 beginners (≤ 1 yr)			sRPE—total weekly	1617 ± 459	1595 ± 446		1716 ± 506			
							Monotony	1.2 ± 0.2	1.2 ± 0.2		1.2 ± 0.2			
							Strain	1920 ± 791	1921 ± 824		2129 ± 944			
							Players vs. experts					*r* = 0.70		
							Players vs. beginners					*r* = 0.72		
							Beginners vs. experts					*r* = 0.80		
Brink et al [24]	16 M (U19/17)	Professional	Soccer	2 coaches	2446 training sessions	6 to 20 Borg scale	RPE	13.6 ± 1.59			14.0 ± 1.72	*r* = 0.24		
				u17 coach (numerous yrs experience)			sRPE	923 ± 207			944 ± 204	*r* = 0.41; *p* < 0.0001		
				u15 coach (numerous yrs experience)										
Viveiros et al [25]	40 M	National Team	Judo	4 separate coaches for the sessions	4 training sessions	CR10	Session 1	4			6.0 ± 0.7			
							Session 2	3			7.6 ± 0.5			
							Session 3	5			5.8 ± 1.6			
							Session 4	3			7.0 ± 0.7			
Wallace et al [26]	12 (6 M, 6 F) 22.3 ± 3.1 yrs	Competitive	Swimmers	2 coaches	20 individual training sessions	CR10	Training duration					*r* = 0.86; *p* < 0.01		
							RPE					*r* = 0.84; *p* < 0.01		
							sRPE load					*r* = 0.85; *p* < 0.01		
Foster et al [27]	6 M	University	Runners	3 coaches but only each athlete had own coach	5 weeks training sessions	CR10	Training duration					*r* = 0.65		
	9 F						RPE					*r* = 0.75		
							sRPE					*r* = 0.74		
							Easy (RPE)	1.8 ± 0.5			2.4 ± 1.4			
							sRPE	91 ± 13			128 ± 72			
							Intermediate (RPE)	3.4 ± 0.7			3.4 ± 1.7			
							srPE	196 ± 66			210 ± 149			
							Hard (RPE)	7.1 ± 1.2			6.2 ± 2.5			
							sRPE	486 ± 194			422 ± 256			

*M*, male; *F*, female, *U*, under; *yrs*, years; *(s)RPE*, (session) rate of perceived exertion; *CR10*, category ratio 10; *RIE*, rate of intended exertion; *ROE*, rate of observed exertion

## Results

### Study Selection and Characteristics

The literature search identified a total of 1615 unique publications, of which 19 studies met the inclusion criteria (Fig. 1). Sixteen studies were included in the forest plot analysis since 3 studies failed to report the correlation between athlete and coach rating of exertion. A total of 252 males and 71 females were observed and 2 studies (56 and 160; total = 216) did not clearly differentiate the sex sample size. Nine studies examined solely males, 1 study included females only, and the remaining 9 studies examined a combination. The age range of participants was from 11 to 24.6 ± 3.8 years old. Seventeen studies analyzed 1 individual sport while 2 studies examined athletes from a variety of sports. The distribution of team sports participation included soccer (5); basketball (3); volleyball (2); hockey (1); netball (1); rugby union (1); and futsal (1), while individual sports were tennis (1); running (2); and swimming (2). Most studies used the Borg CR10 (14), although some also used the 6–20 (5). A total of 52 coaches were included, ranging from 1 to 9 coaches across the different studies, with coaching experience ranging from less than 1 year to > 23 years. Most studies examined the rating of exertion in response to training, ranging from 3 training sessions to a 45-week (full season) period.

### Synthesis of Results

**Fig. 2:**
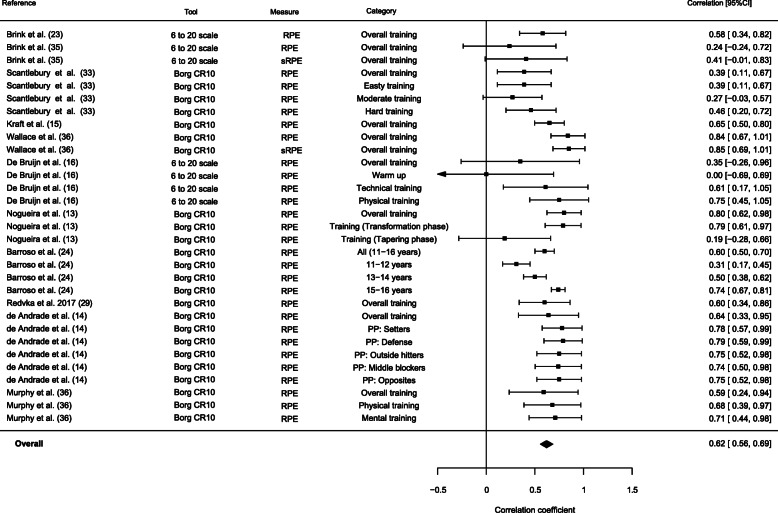
Forest plot of the coach-intended and athlete-perceived rating of exertion

**Fig 3:**
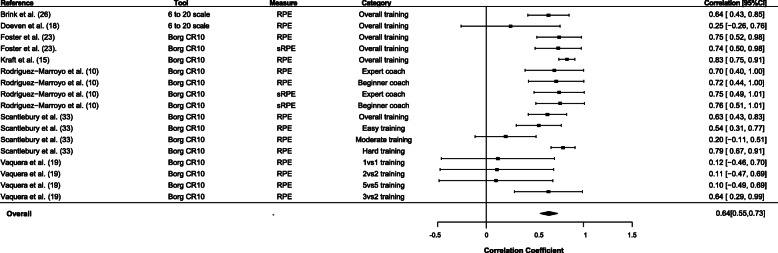
Forest plot for coach-observed and athlete-perceived rating of exertion

A forest plot using a random effects meta-analysis provided the pooled correlation coefficients of coaches’ rating of intended exertion vs. athletes rating of perceived exertion (*r* = 0.62 [95% CI 0.5 to 0.7]) (Fig. 2). The heterogeneity statistics were moderate *I*^2^ = 58.9%, *Q* = 79.9, df(30). Similarly, the random effect meta-analysis of the pooled correlation coefficients of the coach’s ROE vs. the athletes’ RPE is shown in the Fig. 3 forest plot (*r* = 0.6 [95% CI 0.5 to 0.7]). The heterogeneity statistics were moderate with *I*^2^ = 60.7%, *Q* = 40.8, df(16). The funnel plots related to both forest plots did not show any publication bias.

In addition, we also tested for any differences in the pooled correlation coefficients of coaches’ rating of intended exertion vs. athletes’ self-rating of perceived exertion in male athletes as compared with (combined male and female) athletes. The pooled correlation coefficient in studies with male athletes only was (*r* = 0.64 [95% CI 0.5 to 0.7]) compared with studies where it was a mix of male and female athletes (*r* = 0.57 [95% CI 0.5 to 0.7]).

With subgroup analysis, studies that used the Borg CR10 provided a better correlation (*r* = 0.61 [95% CI 0.5 to 0.7]) compared with studies that used the 6 to 20 scale (*r* = 0.47 [95% CI 0.3 to 0.6]). There were also no differences in the correlation coefficients of coaches’ rating of intended exertion vs. athletes self-rating when comparing individual athletes (*r* = 0.64 [95% CI 0.5 to 0.7]) and team athletes (*r* = 0.55 [95% CI 0.5 to 0.6]). Subgroup analysis by sport type revealed that the volleyball players provided the highest correlation coefficient (*r* = 0.75 [95% CI 0.6 to 0.8]).

The random effect meta-analysis of the pooled correlation coefficients of the coaches’ rating of observed exertion vs. athletes’ self-rating of perceived exertion when analyzed using sex as a subgroup revealed that men had a lower correlation coefficient of (*r* = 0.39 [95% CI 0.1 to 0.6] when compared with women (*r* = 0.73 [95% CI 0.5 to 0.9]) and combined men and women athletes (*r* = 0.67 [95% CI 0.5 to 0.8]).

There were very few studies (*n* = 4) that used the 6 to 20 scale and also 2 studies involving individual athletes actually reported the correlation of coaches’ rating of observed exertion vs. athletes’ rating of perceived exertion. Compared with many other sports, basketball athletes provided the lowest correlation coefficient of this relationship (*r* = 0.26 [95% CI − 0.0 to 0.4]). While comparing the rating scale RPE vs. sRPE as subgroups, the correlation of coaches’ rating of observed exertion vs. athletes’ self-rating of perceived exertion was higher when using sRPE (*r* = 0.75 [95% CI 0.5 to 0.9]) compared with RPE (*r* = 0.62 [95% CI 0.5 to 0.7]).

## Discussion

This aim of this systematic review was to examine the association between athletes’ RPE and coaches’ intended RIE and/or ROE. The pooled correlation coefficients of coaches’ rating of intended exertion vs. athletes’ self-rating of perceived exertion were *r* = 0.59, and for coaches’ ROE vs. athletes’ RPE *r* = 0.61. A large variance was observed for the relationship between coach and athlete among the different studies. In what follows, we discuss our findings and draw upon factors that may affect the relationship between athlete and coach perception and what practitioners should be cognizant of that may help refine their approaches to the use of RPE.

### Sport/Training

The physical, cognitive, technical, and tactical demands are highly unique to each individual sport. Accordingly, it would seem much of the coach-athlete mismatch that is observed among studies included in this systematic review may be mediated by the different demands. It could be speculated that individual sports, or those with fewer participants, may provide a slightly better association between the coach and athletes. The notion being that prescribing training for individual sports (i.e., running, swimming) allows the coach to be more vigilant to the workload completed during the session (e.g., control of meters swam/ran, time taken) which will likely facilitate a higher level of agreement between the athlete RPE and coach RIE and/or ROE. While this is theoretically appealing, we have shown that this may not be represented in the results from this review.

Training content for many sessions can often be divided into physical, technical, and tactical components. In the study examining volleyball players and coaches, the authors [23] found differences between expert and beginner coaches’ RPE, compared with athletes’ RPE, but no differences were observed for technical/tactical training and match play. In some instances, the physical element is coordinated and delivered by the fitness coach, during which time the tactical/technical coach may be setting up their own session and/or be involved in discussions with colleagues or other athletes [23]. Conversely, during technical training, coaches’ presence may be prominent and tasked with observing and correcting athletes’ mistakes, which may make them better informed to interpret the exertional demands [22].

### Season and Competition Phase

Training design in team sports involves the manipulation of volume, intensity, and frequency and is often influenced by the training objective and phase of the season. Pre-season is focused on meeting the demands of in-season competition and training during this period has a large focus on recovery from competition and maintenance of fitness levels [30]. In some cases, coaches may be more attentive towards higher self-reported measures of exertion of training and competitions during the pre-season period, as a large focus is on developing fitness. In contrast, the in-season period is more focused on results and outcome(s) of competition, and therefore, information from ratings of exertion may become less important. Andrade Nogueira et al. [19] examined youth swimmers and their coach’s rating of exertion during the last microcycle of the transformation phase (session 1 to 11) and the following mesocycle—the tapering phase (session 12–18). In the transformation phase, athletes tended to overestimate the coach’s RPE (4.2 ± 2.0 vs. 3.9 ± 1.4; *p* = 0.001), while in the Tapering Phase, the RPE tends to be underestimated by the athletes in relation to the coach (2.2 ± 0.8 vs. 2.9 ± 0.3, *p* = 0.001) [20]. Also, in the transformation phase, larger internal load values were observed compared with those in the tapering phase, both in the RPE by the athletes (4.2 ± 0.3 vs. 2.2 ± 0.4. *p* = 0.0001) and the RPE by the coach (3.9 ± 2.0 vs. 2.9 ± 1.4; *p* = 0.001) [20]. Overall, the percentage of agreement was larger in the Transformation Phase (64%) compared with the tapering phase (42%). In the study by Kraft et al. [10], multi-sports were analyzed where it was shown that men’s and women’s basketball were examined at the beginning of the season and soccer and volleyball were observed near the end of their competitive seasons [10] with data collected for only 1–2 weeks. Both of these factors may in part explain the lower correlations they reported, along with the fact they had four different coaches involved and this may explain the variability in the association between sports. This is a consideration for the practitioner in which they may require a more vigilant approach in obtaining a rating of exertion scores during certain phases of the season, possibly the end of some competitive seasons, for example, soccer, where there may be a greater number of matches to contest and a likelihood that subjective ratings of exertion of the athlete and coach may be less aligned. It is also worth highlighting the large variance relating to the time period of data collection we found among the studies, with a range from 3 sessions [12] to 45 weeks (314 sessions) [18] that allowed for data collection. It is plausible that the (in)compatibility between coach and athlete rating of exertion may be higher during shorter observation periods (fewer sessions analyzed) since there is a greater homogeneity compared with a longer period of analyses (seasonal, training phases); however, this requires further investigation.

### Match and/or Training Comparison

Training usually constitutes the greatest proportion of time in a weekly program; however, competition is the most important factor considered when adjusting training load [2]. All the studies meeting our criteria examined the relationship between coach and athlete rating of exertion during training except two studies reporting the association in response to competition, with the number of training sessions included ranging from 9 to 3024 sessions. In one of the studies which examined competition, Doeven [13] reported a low correlation (*r* = 0.25) between coach and athlete reported exertion, lower than the general association seen for training observed studies, suggesting that competition-specific factors may influence RPE. Away from the training/competition facility, the coach will also have little insight into the social activities and demands (travel, business) that players may undertake in the days before and after competition [13]. Though it would be challenging given the chaotic and often sensitive nature of recording rating of exertion around competition time, it would seem fruitful if there were more studies relating to this.

### Exercise Selection/Drill

It is possible that differences may exist not only between sports, but even for different drills and exercises within a chosen sport. The exercise mode and fitness status have been shown to influence RPE scores and may explain differences observed in some studies, For example, De Bruijn et al. [12] reported athletes RPE and physiotherapists RIE for nine amateur soccer players during 4 to 6 months of rehabilitation post-surgery performing on-field training. The author reported that the goal was to increase coordination, balance, and strength, and therefore, focus was not exclusively on increasing cardiovascular capacity. An agreement was shown for the second (pass, run, and dribble exercise (10–15 min) (*r* = 0.61) and the third part of the session (short sprints with small-sided games (5–10 min) (*r* = 0.75), but not for the prior 10–15-min warmup. According to the physiotherapists’ RIE, the warmup was not supposed to be physically demanding, suggesting an underestimation of the load during this phase. From the studies included in the current review, de Bruijn et al. [12] observed a disparity between different drills performed in a rehabilitation setting while Vaquera et al. [16] reported mean differences between coach and basketball players’ perceptions of exertion were 1.13, 1.72, 0.63, and 2.20 AU during 1 vs. 1, 2 vs. 2, 5 vs. 5, and 3 vs. 2 small-sided games, possibly suggesting the number of involved players can contribute to a possible mismatch between coach and athlete rating of exertion [31]. Even the novelty of a given task may affect an individual’s perception of training time [28], which is recognized as a component of the calculated sRPE training load metric.

### Training Classification

Some studies [11, 18] have shown when training sessions are designed to be hard by the coach, these sessions are perceived less intense by the athletes and when designed to be less intense by the coach, the athletes perceive them to be harder. For example, De Andrada [20] showed when the coach intended an easy training session in a group of 15 high-level male volleyball athletes, only 3% of the players perceived it as such, and actually, the vast majority of athletes (90%) reported it as being of moderate intensity. In the sessions proposed as moderate and hard, 68% and 37% of the athletes had the same perception as the coach, while 53% underestimated the sessions’ intensity, classifying it as moderate, with the authors reporting a kappa index of 0.64, for all positions. Interestingly, Barnes et al. [11] showed male and female cross-country athletes rated coach-intended easy sessions significantly harder during each month of the season. Furthermore, men rated moderate-intensity sessions significantly higher than coaches, whereas females rated hard-intensity sessions significantly lower than coaches. There was no difference between males and coach’s hard sessions or females and coach’s moderate sessions.

This large discrepancy between training distribution and classification of intensity seems to be an observation in other studies. For example, of the 34 sessions analyzed, Andrade Nogueira et al. [19] found that 5.9% were classified as easy, 68% as moderate, and 26.5% as hard. While it may allow for a non-polarized approach of training, practitioners should also be cognizant of the potential incidence of training monotony and a narrow range of stimuli which may lead the athletes to suboptimal performance [27]. Indeed, if large amounts of training are consistently performed at a moderate intensity, then athletes may not be exposed to, and therefore unlikely prepared for, the worst-case scenarios associated with competition and may be at a heightened risk of injury. Practically, a mismatch on coach-intended easy sessions may lead to the athlete overtraining if they consistently perceive the session to be harder than intended. Conversely, if the session is perceived to be easier than intended, the athlete may not be exposed to sufficient stimulus to promote adaptation.

### Training Age

Previous training experience and familiarity with load monitoring are likely to be important factors that may influence the relationship between athlete and coach rating of exertion. For example, evidence shows the agreement between coach and athlete rating of exertion increases in accordance with athlete chronological age [21]. In the current review, the group with the youngest athletes (11–12 years old) yielded the lowest correlation coefficient (*r* = .31, *p* < .001), whereas the older group (15–16 years old) presented the highest correlation (*r* = .74, *p* < .001). A greater training exposure may allow individuals to more easily identify intensity levels by allowing athletes to experience and recognize a variety of physiological changes (e.g., heart rate, ventilation, oxygen uptake, blood lactate), thus creating an internal anchoring for their effort [32].

### Fitness and Recovery

Brink [17] aimed to explain a potential mismatch through on-field training characteristics, intermittent endurance capacity, and maturity status with the former shown to be a positive predictor of coaches’ intended and observed rating of exertion. Coaches may consider that players with a lower intermittent endurance capacity will perceive the training as harder [17] and indeed, previous research has shown that cardiorespiratory fitness may influence the physiological responses at a given RPE [33]. Practically, coaches are unlikely and/or willing to report ratings of observed exertion for all their athletes, rather than taking a more global viewpoint of the collective group. There may also be instances where certain athletes are notably identified as “under- or overexerting.” Performance information from fitness test results, past achievements, or body language (increased perspiration, body language) during training or competition may actually influence the coaches’ observed rating of exertion.

A better understanding of the athlete’s level of recovery, as a mediator for the RPE mismatch is another justification to record competition exertion. Doeven [13] also examined the total quality of recovery (TQR) of 14 professional basketball players during an in-season 6-week phase. Participants reported their TQR score before, and RPE after training sessions while coach ROE and observed recovery (TQ-OR) of the players were also recorded. Correlations between coach- and player-perceived exertion and recovery were *r* = 0.25 and *r* = 0.21, respectively. While players’ RPE were lower than coaches’ ROE (15.6 ± 2.3 and 16.1 ± 1.4; *p* = .029), it is the difference between TQR and TQ-OR (12.7 ± 3.0 and 15.3 ± 1.3; *p* < .001) that may be of greater importance as the within-day association was *r* = .68 and non-existent after 1–2 days [13].

Though athletes’ RPE and their pre-training perceived level of wellness may not be closely related, it is an important consideration for examining the possible mismatch that occurs between athlete and coach. In a separate study, Kraft et al. [10] showed an overall correlation of *r* = 0.25 (range *r* = 0.13 to 0.38) between coaches and athletes’ perception of athlete recovery (using the perceived recovery status scale), across different sports (volleyball, soccer, basketball). Practically, a mismatch as evidenced by a higher rating of perceived recovery values may have led to the coach overestimating the difficulty of a task in relation to external cues (i.e., exterior signs of athlete effort may have been misinterpreted due to an incorrect notion of recovery) [10]. Subsequently, it is important that the coach has a good understanding of athletes’ perceived level of recovery since this will likely affect the association between coach and athlete rating of exertion.

### Coaching Experience

In one study included in this review, the response for expert (more than 23 years) and beginner (less than 1 year) coaches’ RIE was compared in a sample of 18- to 25-year-old female volleyball players. Beginner and expert coaches’ RIE were shown to be strongly correlated (*r* = 0.90, ICC = 0.90) as well as eliciting a similar response to the athletes’ RPE (expert, *r* = 0.75, ICC = 0.80; beginner, *r* = 0.76, ICC = 0.83) [23]. Coaching practice is heavily influenced by individual experience, tradition, emulation, and historical precedence [34]. Since player experience seems to influence athletes’ RPE [21], it is plausible that experience is also a factor in coach-intended and observed rating of exertion. Coaches’ experience with a specific individual or group of athletes should allow for familiarization and a better understanding of those individuals which may manifest in better relations and thus, a better understanding of the athlete’s perceptions of exertion. While amassing years of coaching experience is often recognized as a testament to a good coach, it would appear that it may not provide any better indication of the athlete’s perception of exertion. It could be speculated that beginner coaches are less experienced in terms of training/competition, they may be just as attuned with the athlete’s subjective feelings, mediated by a similar age group, or less influenced by those past training experiences.

### Co-observer

Since an athlete’s RPE response may be influenced by the presence of a co-observer (i.e., another athlete/player being present), it is reasonable to suggest that the presence of a colleague from the support staff may also impact upon the coaches’ RIE and/or ROE. In the study by Redvka [14], the members of the coaching team responsible for the training session, technical or fitness coach, rated the RPE in accordance with “the main objective” determined by the technical soccer coach for the training session. Regarding the types of training proposed for the preseason period, differences were found in relation to time for tactical training in relation to physical training, yet no differences were observed when comparing the S-RPE prescribed by coaches and perceived by soccer players (*p* > .05) in the different proposed training types. Though the authors acknowledged that this was performed without interference from the researchers, this process is likely a more realistic representation of the applied environment. In many sports, the coach may meet daily with their support team to discuss the training objective and their roles for the forthcoming session. While the coach is in charge of the overall session it is typical to relinquish parts to other coaches, for instance, the physical aspect may be coordinated by the fitness coach. Recognizing that rating of RIE and/or ROE is unlikely to be solely provided by the coach with no influence from external bodies is worth considering when practitioners record these data.

### Tool Used

The Borg CR10 is the most commonly applied tool for examining the association between coach and athlete ratings of exertion. Most studies in the current review have solely used RPE, despite sRPE being a more common tool in the applied setting. While comparing the rating scale RPE vs. sRPE as subgroups, the correlation of coaches’ rating of observed exertion vs. athletes’ rating of perceived exertion was higher when using sRPE compared with RPE. Therefore, factoring possible constructs such as duration of session are likely important when examining the relationship between coach and athlete perception of exertion. There were very few studies (*n* = 4) that used the 6 to 20 scale and also 2 studies involving individual athletes actually reported the correlation of coaches’ rating of observed exertion vs. athletes’ rating of perceived exertion.

Differential RPE (breathlessness sRPE-B, leginess sRPE-L) is also worth investigating as it has been proposed as providing more precise detail for the athlete and coach [35], though further research is required to examine this response and the relationship between coach and athlete perceptions of exertion. Distinguishing between skills, physical, and cognitive RPE has gained some acceptance as a viable method; hence, a possible advancement may be to record separate technical and non-technical training sRPE scores and differentiate between each in future analyses. However, the level of evidence for idiosyncratic acute and chronic periods and distinguishing between technical and non-technical training sRPE in training load models is not yet well developed. Some studies have analyzed the physical, technical, and tactical components of training which may prove useful to establish where possible (in)compatibility may exist. From the current review, Murphy et al. [22] separated training into the physical and mental exertion aspect in a group of elite junior tennis players, with the correlation between the coach and athlete shown to be similar for the mental (*r* = 0.71) and physical component (*r* = 0.69). Since many sports comprise multiple drills and focus on different components of the sport, it is important that the coaches, practitioners (sports scientists, fitness coaches), and athletes are aware of the loading subtleties of drills throughout the sessions.

## Limitations

Overall, studies have been limited in their reporting of athlete and coach characteristics. Notably, little is known regarding the detailed coaching experience or the period of familiarization of the tool for the athlete as well as the coach with exposure ranging from a few days to several years, demonstrating a possible variance of exposure. Contemporary RPE monitoring in most sports environments is often one component of monitoring, which includes objective (e.g., Global Positioning Systems) and subjective monitoring (e.g., perceptions of wellness and recovery) as well as physical fitness data. Unfortunately, there were no studies that provided a complete analysis of the above information over an extended period of time. We believe that doing so would provide a more accurate representation of current monitoring in sports. Understandably, a practical issue in such coach-related research is the unfavorable coach-player ratio which may restrict generalizability to other coaches especially with the number of coaches involved between studies differing quite largely. Moreover, 8 of the 16 studies included merged both male and female, and as such, findings should be carefully interpreted while comparisons are difficult since the experimental designs have differed among studies. Inconsistencies between the athlete and coach may be a result of the instructions and protocol used. This has varied from “if a friend who did not understand the specific training expressions of athletics were to ask you how hard your training session was, how would you reply?” [27] to “how was your session?” [14]. Albeit possibly subtle, a difference in the wording and how the question is presented may influence participant’s perception and response and requires recognition in terms of the importance of appropriate communication and cueing. While it is unknown what effect, if any, this may have on the competence/accuracy of a coach’s use of the tool it does make comparisons among studies difficult.

We acknowledge that the exclusion of non-English articles may be considered a source of bias and recognize that some inferences may be challenging since pooled correlation coefficients may inflate the degrees of freedom in analysis which may lead to imprecise analysis. As part of the review we included analysis of different tools which may not be appropriate when making statistical inferences; however, we feel that it provides a valid insight into the realities of variance among daily monitoring across sports. Also, we acknowledge that in accordance with Cochrane risk of bias assessment and the resultant difficulty in randomly selecting participants in these studies, there was a risk of bias associated with these analyses; however, we feel that the findings of our results should still be recognized as being impactful for the practitioner as well as highlighting a need for further research with refined methodological designs.

## Practical Application and Suggestions

There are likely to be several unwanted implications of a consistent mismatch, namely the sustenance of a coaching context that jeopardizes the physical, mental, and emotional health of the athlete. Chronic exposure to inappropriate training load can result in increased likelihood of overtraining/injury or under prepared athletes. While there are reports that coaches believe no system can replace their judgment and personal interaction with each athlete [36], the findings from the current review shows that the coach’s judgment may not be cognizant with those of the athlete. Therefore, practitioners should be wary of a possible disparity between coach and athlete rating of exertion due to the heightened potential of unfavorable adaptations.

Education is often proposed as a strategy to improve compliance with athlete monitoring. An interesting study by Brink [9] examined the effects of coach feedback on RIE and ROE in association with a group of soccer players. Although the agreement between ROE and RPE improved following feedback (F(1.229) = 9.16, *p* = 0.003, ES 0.4), this was not the case for RIE. The feedback was shown to improve the agreement more for the hard sessions (*p* < 0.004, effect size 0.6) but not easy and intermediate sessions. Interestingly, it is also worth noting that while the feedback improved agreement between RIE and RPE for eight participants, there was also a larger discrepancy for the other four individuals. Therefore, while educating staff is often considered an important factor for any aspect of work, it is not always clear as to the best ways that this may be achieved. Scantlebury et al. [15] showed that even though the coach modifies their intended RPE following training, the observed RPE still moderately underestimates RPE for easy session with a small overestimation of athlete RPE for intended moderate sessions.

Athlete and coach education and improved communication are suggested to be important elements of load monitoring. While this may theoretically sound appealing, there is a general lack of understanding of appropriate strategies for implementation and examples of what this entails. Since feedback to the coach may improve most, but not all, scorings of RPE, it should be recognized that further work is required in this area to better align load monitoring practices between athlete and coach.

The current state of daily monitoring may be a paradigm that requires careful consideration, particularly with the emergence of different surveillance technologies. While asking athletes daily “how intense do you consider that session” may seem innocuous to many practitioners, it should be remembered that this is often coupled with several other monitoring strategies. During times of high competition stress, the cumulative effects of these approaches may be considered a psychological burden to athletes that may present itself in a suboptimal interaction with the question being asked.

It may be that for some practitioners once a load monitoring assessment tool has been included as part of a daily protocol then this needs to remain stringent practice. Taking this away or modifying the tool application may often be feared as it may present itself in missing an important part of the fitness or injury “puzzle.” Therefore, while the rating of perceived exertion is considered reliable, valid, and sensitive, it may not always be a*ppropriate*, and while collecting these data can be useful, it should not easily be assumed to be *impactful* across the full spectrum of physical, psychological, social, and emotional care for the athlete and coach.

## Conclusion

The purpose of the current review was to summarize the available evidence examining the association between the athlete’s rating of perceived exertion and coach’s intended and/or coach observed rating of exertion. Though the RPE is considered a popular tool, we have identified coaches and athletes may not always have full agreement, meaning that consideration should be given to factors that may mediate any possible disassociation. Overall, we found a moderate to strong relationship between the coach’s RIE and athletes’ actual RPE while this was only slightly improved for coach’s ROE and RPE. Factors affecting the strength of these relationships may be dependent on the athlete, the coach, their relationship, or contextual environmental factors. In this review, we have provided an insight into considerations for the practitioner to understand contextual factors that may impact the athletes’ reporting of perceived exertion. By considering the factors impacting the relationship between coach and athlete rating of exertion, the practitioner may be able to better assist in the monitoring and interpretation of training and match load.

## Data Availability

Upon request to the author.

## Abbreviations

RPE: Rate of perceived exertion
RIE: Rate of intended exertion
ROE: Rate of observed exertion
sRPE: Session rating perceived exertion
QAREL: Quality appraisal for reliability studies
PRISMA: Preferred Reporting Items for Systematic Reviews and Meta-Analyses
CI: Confidence interval
TQR: Total quality of recovery
TQ-OR: Total quality of observed recovery
